# Vitamin D: An Essential Nutrient in the Dual Relationship between Autoimmune Thyroid Diseases and Celiac Disease—A Comprehensive Review

**DOI:** 10.3390/nu16111762

**Published:** 2024-06-04

**Authors:** Francesca Gorini, Alessandro Tonacci

**Affiliations:** Institute of Clinical Physiology, National Research Council, 56124 Pisa, Italy; alessandro.tonacci@cnr.it

**Keywords:** vitamin D, autoimmune thyroid disease, celiac disease, immune system, gut microbiota, AI

## Abstract

Autoimmune thyroid diseases (AITD) are among the most frequent autoimmune disorders, with a multifactorial etiology in which both genetic and environmental determinants are probably involved. Celiac disease (CeD) also represents a public concern, given its increasing prevalence due to the recent improvement of screening programs, leading to the detection of silent subtypes. The two conditions may be closely associated due to common risk factors, including genetic setting, changes in the composition and diversity of the gut microbiota, and deficiency of nutrients like vitamin D. This comprehensive review discussed the current evidence on the pivotal role of vitamin D in modulating both gut microbiota dysbiosis and immune system dysfunction, shedding light on the possible relevance of an adequate intake of this nutrient in the primary prevention of AITD and CeD. While future technology-based strategies for proper vitamin D supplementation could be attractive in the context of personalized medicine, several issues remain to be defined, including standardized assays for vitamin D determination, timely recommendations on vitamin D intake for immune system functioning, and longitudinal studies and randomized controlled trials to definitely establish a causal relationship between serum vitamin D levels and the onset of AITD and CeD.

## 1. Introduction

Autoimmune thyroid diseases (AITD), along with type 1 diabetes, are currently the most frequent autoimmune disorders, globally affecting around 3–5% of the general population [1,2], and with a predominant prevalence in females in both pediatrics and adulthood [3,4]. Although AITD embrace a wide range of phenotypes, the most common conditions include Graves’ disease (GD) and Hashimoto’s thyroiditis (HT), both of which are characterized by lymphocytic infiltration of the thyroid parenchyma and also major causes of thyroid gland dysfunctions (hyperthyroidism and hypothyroidism in iodine-sufficient areas, respectively) [4,5]. Despite large variability by geographic region, based on a recent meta-analysis involving 19 countries and 22,680,155 participants, the overall prevalence of HT in adults has been estimated at 7.5% globally, with a four-fold risk in women compared to men [6]. The population prevalence of GD ranges between 1 and 1.5%, with 3% and 0.5% of diseases affecting women and men, respectively, and a maximum incidence in patients aged 30–60 years [7]. Loss of immune tolerance, reactivity to thyroid autoantigens—circulating thyroid peroxidase (TPO) and thyroglobulin (TG) antibodies (Ab), mainly in HT, and thyroid-stimulating hormone receptor (TSHR)Ab as the hallmark of GD—and infiltration of the thyroid by T and B lymphocytes, which ultimately drive the cytokine production, are the major determinants of AITD [8,9]. Furthermore, although tissue injury in AITD results from both cell-mediated and humoral responses, while the autoimmune reaction in GD is triggered by an increase in the number of CD4+ T cells, the infiltration of CD8+ T cells with cytotoxic properties is a characteristic of HT [10,11]. The mechanisms underlying the autoimmune attack in AITD probably entail an interaction between genetic susceptibility (i.e., the presence of specific genes significantly associated with AITD occurrence) and environmental factors (e.g., radiation exposure, iodine intake, smoking habits, drug use, virus infections) [5]. On the other hand, accumulating evidence indicates that modifications in the gut microbiota composition, i.e., a large number of microorganisms in the human gastrointestinal tract forming a symbiotic relationship with the host, have been related to the onset of numerous autoimmune disorders, such as type I diabetes, rheumatoid arthritis, systemic lupus erythematosus, inflammatory bowel disease, Behçet’s disease, autoimmune skin conditions, and autoimmune neurological diseases, which also supports a possible role for microbiota alterations in the pathogenesis and progression of AITD [12,13]. Additionally, there is also a close association between thyroid hormone and the intestine in physiological conditions since the intestine is one of the target organs of triiodothyronine, with the gut microbiota pivotal in the homeostasis of thyroid function, as it influences iodothyronine synthesis, conversion, and storage, as well as through immune regulation [14].

Furthermore, the gut microbiota has been associated with many intestinal disorders, including celiac disease (CeD), intestinal bowel diseases, irritable bowel syndrome, colorectal cancer, chronic liver diseases, and pancreatic disorders [15,16,17]. CeD is an autoimmune enteropathy induced by dietary gluten in genetically susceptible individuals featuring chronic inflammation, crypt hyperplasia, and villous atrophy of the small intestine, which may cause a plethora of clinical manifestations like chronic diarrhea, weight loss, iron deficiency, bloating, constipation, chronic fatigue, headache, abdominal pain, and osteoporosis, as well as a completely silent disease [18,19,20,21]. If CeD was originally considered a rare disorder, over the last decades, the implementation of more sensitive and specific detection programs has allowed the discovery of subjects without symptomatic presentation [20]. CeD currently represents a serious issue of public health, with a worldwide prevalence of around 1.4% based on serological tests and 0.7% based on biopsy results, albeit variations may occur by sex, age, and country of origin [21,22]. CeD has been linked to numerous immune diseases, both inside (e.g., ulcerative colitis, Crohn’s disease, autoimmune liver disease) and outside the gastrointestinal system (e.g., immunoglobulin (Ig)A nephropathy, pernicious anemia, Addison’s disease, type 1 diabetes mellitus, Sjögren’s syndrome) [19,23]. As concerns the association between CeD and AITD, it appears to be bidirectional, with a mounting prevalence of AITD among celiac patients and vice versa [9]. Various factors such as common genetic features, changes in the composition and diversity of the gut microbiota, and the deficiency of nutrients like vitamin D, essential for both the thyroid and the immune system, the regulation of which leads to intestinal modifications capable of increasing the risk of autoimmune diseases, may likely contribute to this dual relationship [24,25]. In fact, vitamin D is not only a pro-hormone that regulates calcium and phosphorus homeostasis and bone health, but, recently, it has been recognized as exerting several extra-skeletal functions, including influencing the development, clinical course, and treatment of various autoimmune disorders [24,26,27,28].

Under such premises, taking into account the quick advancement of the related literature and the lack of recent related papers on this topic, the purpose of this comprehensive review is to summarize the current state of knowledge dealing with the relationship between AITD and CeD and the mechanisms underlying this interplay, also highlighting the potentially relevant role of vitamin D as risk factor of these two conditions in case of deficiency and, at the same time, as a therapeutic option, in the form of a supplement, in their treatment. The outlook about possible future technology-based strategies for vitamin D supplementation is also presented.

## 2. Vitamin D: The General Features

In recent decades, it has been recognized that vitamin D, belonging to the family of steroid hormones, beyond maintaining and protecting the skeletal system through the regulation of circulating levels of calcium, magnesium, and phosphate, exerts various extra-skeletal effects, including a key role in the immune system [25,29,30]. The two major forms include vitamin D, cholecalciferol, known as vitamin D3 and mainly contained in animal foods (oily fish, such as sardines, herring, tuna, mackerel, salmon, and cod liver oil, egg yolks, liver or organ meats), and ergocalciferol, or vitamin D2, found in some mushrooms and also used as a dietary supplement [31]. There are also commercial foods fortified with vitamin D (e.g., dairy products, soy milk, and cereals), especially suitable for infants and children to challenge vitamin D malnutrition and related disorders, although most vitamin D, in the D3 form, is synthesized at the skin level from 7-dehydrocholesterol (7-DHC), a precursor of cholesterol, after exposure to ultraviolet (UV) light [31,32,33,34,35,36]. Further factors, such as genetic polymorphisms, aging, race, skin color, body mass index (BMI) and obesity, sunscreen use, latitude, time of day, and season, along with type, dose, and duration of exposure to sunlight, may influence vitamin D synthesis [37]. Upon UV exposure, vitamin D undergoes two successive hydroxylation phases to become the active hormone: the first mainly in the liver, where vitamin D 25-hydroxylase synthesizes 25-hydroxyvitamin D [25(OH)D3], namely calcidiol or calcifediol, and the second in the proximal tubules of the kidney (with local production also occurring in the skin) [38] by the action of 1-hydroxylase encoded by the *CYP27B1* gene, the rate-limiting enzyme, to produce 1,α,25-dihydroxyvitamin D [1,25(OH)_2_D3] or calcitriol [33,39]. Although 25(OH)D3-1α-hydroxylase is expressed in several other cell types (keratinocytes, brain glial cells, monocytes, and parathyroid cells), probably for autocrine/paracrine purposes, the kidney is the only organ that produces and releases calcitriol into the circulation, where, with a half-life of 20–24 days, it represents the main indicator for determining the status of vitamin D [24,40]. On the other hand, despite very low serum levels strictly regulated by parathyroid hormone (PTH), calcium, and phosphate and a half-life of 3–6 h, 1,25(OH)_2_D3 is the active form of vitamin D, which, by binding to the nuclear vitamin D receptor (VDR), has direct effects on the genome [38,41,42,43]. The receptor is widely distributed in various body tissues and cells (e.g., renal tubules, parathyroid and pituitary gland cells, skin keratinocytes, breast epithelium, beta islet cells in the pancreas, osteoblasts and chondrocytes in bones, monocytes, macrophages, and T lymphocytes), although the intestine is characterized by the highest expression of VDR [43]. The bond to 1,25(OH)_2_D3 allows VDR to reach the nucleus and form the VDR:VDR complex or a heterodimer with retinoid X receptor, which interacts with gene response elements in the promoter regions of target genes, thereby modulating the expression of more than 1000 genes in humans, corresponding to approximately 3% of the human genome [33,40,44,45]. In addition to genomic activity, both calcitriol and calcifediol can promote non-genomic effects on target cells via the membrane isoform of VDR [40,46].

Notably, there is an alternative metabolic pathway to produce the active hormone involving CYP11A that catalyzes the conversion of vitamin D3 to 20-hydroxyvitamin D3 [20(OH)D3] (this product can be further modified by other CYP enzymes such as CYP2R1, CYP27A1 and CYP27B), 22(OH)D3 and other di-hydroxy (e.g., 20,23(OH)2D3) and tri-hydroxy species) without exerting any catalytic activity on 25(OH)D3 [47,48,49]. These hydroxymetabolites, although produced in the skin, are detectable on the systemic level in the human serum, skin, and placenta; in the pig adrenal gland; and even in natural products like honey [50,51,52]. 20(OH)D3 and its hydroxylated derivatives may accumulate in the human epidermis, where they show potent biological activities, exerting anti-inflammatory, anti-tumor, and photoprotective effects through mechanisms of actions that include, along with VDR, aryl hydrocarbon (AhR), liver X (LXRα and β) receptors (agonistic effect), and retinoic acid orphan receptors (antagonistic effect) [50,53,54].

Within the noncanonical pathways of vitamin D activation, it is important to say that the UVB-induced photolysis of the B-ring of 7-DHC and other 5,7-dien-3β-ols can give rise to various secosteroids, including lumisterol and tachysterol, measured in both serum and epidermis in humans, which are converted by CYP11A1 and CYP27A1 to pre-vitamin D3 photoproducts, producing 20-hydroxylumisterol3 [20(OH)L3], 22(OH)L3, 20,22(OH)2L3 20S-hydroxytachysterol3 [20S(OH)T3] and 25(OH)T3 [50,53,55,56]. Ex vivo and in vivo experimental studies in mammals have documented that secosteroids have photoprotective, antioxidative, and pro-differentiation properties dependent on mechanisms based on the binding to nuclear receptors. In particular, recent studies have shown that, like the hydroxyderivatives of 20(OH)D3, lumisterol and tachysterol metabolites can bind, in addition to the non-genomic VDR site, to AhR, LXRs, and peroxisome proliferator-activated receptor gamma (PPARγ), suggesting the potential of these products to play a pivotal role not only in the skin but also in other organs that present these receptors [50,55]. Importantly, if vitamin D is ubiquitous in all organisms and ergosterol and other 5,7-dien sterols are produced by several eukaryotic unicellular organisms, insect species, although they contain melatonin metabolites (also detectable in bee honey), are unable to produce sterols endogenously, paving the path to the future search for different secosteroids with high biological power in other organisms [56].

A large amount of genetic, molecular, cellular, and animal studies widely suggest that vitamin D status can be related to cell proliferation; inflammation; cancer; autoimmune, cardiovascular, and neurological diseases; metabolic syndrome; and mortality [28,44]. Nonetheless, to date, serum concentrations of 25(OH)D3 associated with deficiency and consequent increased risk of rickets and osteomalacia, adequacy for bone health, and overall health have not been fully identified. Based on the Endocrine Society guidelines, vitamin D deficiency corresponds to serum 25(OH)D3 levels below 20 ng/mL (50 nmol/L), while vitamin D insufficiency has been set at 21–29 ng/mL (52.5–72.5 nmol/L) [43,46]. Therefore, a serum concentration above 30 ng/mL (75 nmol/L) is recommended for both children and adults, including pregnant and lactating women, to maintain good skeletal health [57]. The European Food Safety Authority (EFSA) has established vitamin D sufficiency at a serum 25(OH)D3 concentration of 20 ng/mL for adults [58], in line with the Recommended Dietary Allowances (covering requirements of ≥97.5% of the population) of the Institute of Medicine (IOM) [59]. According to the IOM and the Endocrine Society, a daily supplementation of 400 IU/day (10 µg) of vitamin D in all infants from birth to 12 months of age and at least 600 IU/day (15 μg) in infants aged over 12 months and in adults up to 70 years of age are enough to prevent or treat vitamin D deficiency [57,59]. Moreover, while IOM recommended 800 IU/day for adults over 70 years of age, the Endocrine Society proposed a daily intake two to three times greater for adults with BMI > 30 compared to adults of normal weight [60]. EFSA also indicated an adequate intake of 15 μg/day for both children aged 1–17 years and adults, whereas an adequate intake was set at 10 μg/day for infants aged 7–11 months [58] (Table 1). Of note, 25(OH)D3 levels as low as 100 nmol/L appear to be sufficient for all health outcomes, including bone health [61]. In addition, conversely to previous recommendations that set tolerable upper limits for vitamin D below 4000 IU/day for all persons older than 8 years and lower cut-offs for infants and children up to 8 years of age [57,60], a double-blind, randomized clinical trial recently demonstrated the long-term safety of vitamin D even at a dose of 10,000 IU/day [62].

The large variability of currently available analytical assays affects the accuracy of serum 25(OH)D3 measurements and comparisons between studies [24]. While immunoassays (radioimmunoassays, enzyme-linked immunosorbent assays, chemiluminescence assays) are still mostly popular, they present several limitations, e.g., matrix effects, poor antibody specificity, cross-reactivity with other 25(OH)D3 metabolites, and limited release of vitamin D from carrier proteins. On the other hand, liquid chromatography–mass spectrometry, which currently represents the gold standard for the quantitative determination of 25(OH)D3 thanks to its greater precision over a large concentration range and the ability to identify multiple vitamin D metabolites, is still highly costly and time-consuming and also requires well-trained, highly specialized staff [37,63,64].

In the following sections, we will describe the role of the gut microbiota and the immune system in the pathogenesis of AITD and CeD and discuss how vitamin D might intervene in maintaining the homeostasis of these two relevant components and how its levels may be associated with the occurrence of such diseases.

## 3. The Gut Microbiota: Characteristics and Main Functions

The human microbiome is a complex aggregate of all microorganisms residing at various sites in the human body [65], with the term “microbiome” accounting for all microbiota-related genomes and estimated to encode approximately 10 times more genes than the whole human genome [66,67]. The gut microbiota, composed of over 100 trillion microorganisms, mainly complex bacteria and a small amount of viruses and fungi, is implicated in maintaining the host’s state of health [14]. Any compositional and functional alterations of the gut microflora, namely dysbiosis, resulting in an imbalance of pathogenic and protective microbes in the host [68], is involved in the breakdown of this mutualistic relationship and, consequently, in the onset or worsening of pathologies internal and external to the gastrointestinal tract [69,70,71,72]. The intestinal microbiota is dominated by anaerobic bacteria, including the phyla of Firmicutes, Bacteroidetes, Actinobacteria, Proteobacteria, and Verrucomicrobia, with Bacteroidetes and Firmicutes representing 90% of its total amount [16,73]. Thanks to significant advances in the technology of diagnostic methods (i.e., 16S rRNA sequencing on fecal samples), the role of the intestinal microbiota is expanding [14]. In addition to functions related to the control of gastrointestinal homeostasis, metabolism, detoxification, and vitamin synthesis, the gut microbiota has a key role in the development of the lymphoid system, 70% of which constitutes the gut-associated lymphoid tissue (GALT), including macrophages, dendritic cells (DCs), T and B cells, and natural killer cells [70,74]. In healthy conditions, the microbiota has a symbiotic relationship with the host and preserves immune homeostasis through the activation of pattern recognition receptor/pathogen-associated molecular patterns (PRR–PAMP) [75]. As part of PRR–PAMP recognition, the endotoxins lipopolysaccharides (LPS) retrieved on the cell membrane of Gram-negative bacteria and other PAMPs activate PRRs (e.g., Toll-like receptors (TLRs), nucleotide-binding oligomerization domain leucine-rich repeat-containing receptors (NOD), retinoic acid-inducible gene 1-like receptors, and the C-type lectin receptors) expressed by immune cells and triggering both the innate and adaptive immune responses, thus limiting the colonization of pathobionts (opportunistic microbes whose proliferation derive from the imbalance in the homeostasis of symbiotic communities) [16,75,76]. Furthermore, gut microbes are capable of producing short-chain fatty acids (SCFAs, e.g., acetate, butyrate, and propionate) from non-digestible dietary fibers, which, serving as an energy source for colon epithelial cells, contribute to the microbial proliferation and the integrity of the intestinal barrier [16]. SCFAs, especially butyrate, are known to maintain anaerobic conditions in the colon by activating the β-oxidation in the mitochondria and to reduce the availability of nitrate for specific pathogens by binding to PPARγ, in turn inhibiting inducible nitric oxide synthase [16]. Furthermore, the microbiota is involved in the modulation of epigenetic changes, with SCFAs capable of inhibiting histone deacetylases, which results in anti-inflammatory effects, with an increase in naïve CD4+ T cells and circulating regulatory T cells (Tregs), enhanced production of interleukin (IL)-10 in macrophages to regulate T-cell activity in other tissues, inhibition of tumor necrosis factor alpha (TNF-α) secretion, and nuclear factor kappa-light-chain-enhancer of activated B cell (NF-κB) activity [39,77,78].

### 3.1. Gut Microbiota and Autoimmune Thyroid Diseases: Underlying Mechanisms

**Figure 1:**
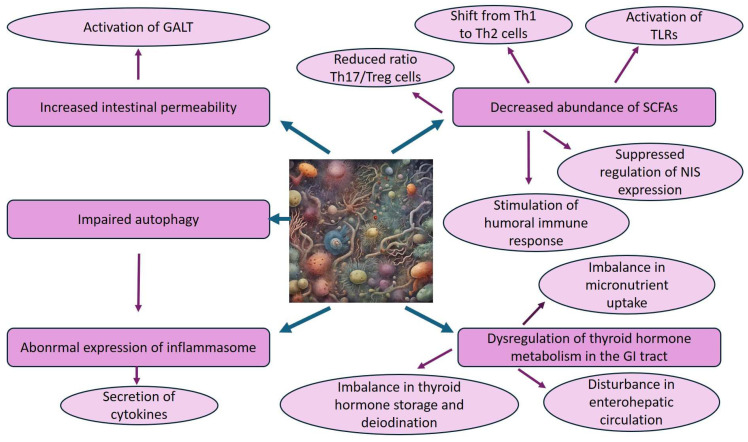
Mechanisms underlying the association between dysbiosis of the gut microbiota and the onset of autoimmune thyroid diseases. Abbreviations: GALT: gut-associated lymphoid tissue; GI: gastrointestinal; NIS: sodium/iodine symporter; SCFAs: short-chain fatty acids; Th cells: T helper cells; TLR: Toll-like receptors; Treg: regulatory T cells.

Although AITD pathogenesis probably occurs due to an interplay between endogenous and exogenous factors, including genetic susceptibility, environmental factors, and autoimmunity, the recognized existence of a gut–thyroid axis and the gut as a relevant endocrine organ led to the hypothesis of a correlation between intestinal microorganisms and AITD [75,79]. Variations in circulating levels of thyroid hormones can be responsible for gastrointestinal disturbance; in particular, the history of overt hypothyroidism has been associated with bacterial overgrowth development in the small intestine, which in turn promotes clinical gastrointestinal symptoms [75,80]. On the other hand, in genetically susceptible subjects, perturbations of the gut microbiota composition or functionality by environmental factors (e.g., long-term diet changes, lifestyle, physical activity, drug therapies, diseases) contribute to the failure of immune tolerance and to the development of a number of immune-mediated disorders, including AITD [12,81,82]. In this framework, several pathogenetic processes have been proposed (Figure 1):

1. An increase in intestinal permeability (i.e., reduction in microvillus thickness and increase in the space between adjacent microvilli), allowing the transit of toxins, antigens, or bacterial metabolites from the gut to the bloodstream, gives rise to the so-called “leaky gut” [83,84]. Decreased integrity of the intestinal barrier promotes the translocation of antigens located into the intestinal lumen to activate the GALT via molecular mimicry. This mechanism, which involves plasma cells generating antibodies directed against antigens expressed on thyroid follicle cells, as well as autoantibodies generated by posttranslational protein modifications, transforms a defensive immune response into autoimmunity due to the structural similarity between microorganisms and host antigens [12,85,86,87].

2. Decreased abundance of SCFA-producing bacteria may cause a number of effects: (a) it suppresses the regulation of the sodium/iodine symporter (NIS) expression, the essential plasma membrane protein that mediates active iodide transport into the thyroid gland for thyroid hormone biosynthesis; (b) it reduces the balance ratio between the two main effectors in CD4^+^ T cells, namely T regulatory (Treg) cells, producing anti-inflammatory cytokines IL-10, transforming growth factor beta 1 (TGF-β1), and promoting tolerance to self and pathogen antigens, and T helper (Th) 17 cells, releasing pro-inflammatory cytokines IL-17 and playing a substantial role in autoimmune diseases; (c) it induces the shift of Th1 to Th2 cells, thus stimulating humoral immune responses against extracellular pathogens; and (d) it activates TLRs, in turn also expressed in thyroid follicular cells in response to various PAMPs, resulting in disruption of immune homeostasis and prolonged production of pro-inflammatory cytokines and chemokines [12,75,88,89,90,91]. In particular, the bond of LPS to TLR-4 triggers innate immune responses through the two major inflammation-related signaling pathways in cells, NF-κB and mitogen-activated protein kinase (MAPK), which activate the synthesis of the pro-inflammatory IL-6, associated with autoimmune disease development [92].

3. The intestinal microbiota can also activate the abnormal expression of the inflammasome, an intracellular multiprotein complex (e.g., NLRP1, NLRP3, NLRC4, AIM2) that, through LPS and bacterial antigens, recruits the adaptor protein ASC and activates caspase-1 to foster the promotion of cleavage and secretion of IL-β and IL-18. Cytokines, in turn, activate neutrophils and DCs, trigger differentiation of Th17 and Tregs, stimulate intestinal epithelial cells to produce antimicrobial peptides, and induce the production of interferon gamma (IFN-γ) [75]. Notably, Guo et al. [93] found an increased expression of NLRP1, NLRP3, NLRC4, AIM2, caspase-1, and precursors of IL-β and IL-18 in thyroid tissues of patients with HT, the first to demonstrate an association between inflammasome and the pathogenesis of HT.

4. Autophagy, consisting of highly conserved catabolic processes by which cytoplasmic material is delivered into the lysosome for degradation, helps to maintain gut homeostasis [74,94]. Conversely, impaired autophagy promotes a pro-inflammatory condition through inflammasome activation, resulting in the deterioration of the intestinal microenvironment and in the imbalance between harmful and beneficial gut bacteria [39,67]. Indeed, in an epithelial-specific intestinal autophagy-related 5 (Atg5) knockout mouse model, Yang and co-authors [95] observed a significant alteration and decreased diversity in the gut microbiota of Atg5-deficient mice compared to that of wild-type mice. Of note, IL-23, essential for the development of autoimmune diseases in several models, contributes to the suppression of autophagy and the accumulation of reactive oxygen species (ROS) in thyroid follicular cells in HT pathogenesis through the activation of the AKT/mTOR/NF-κB signaling pathway [96].

5. Dysbiosis may lead to disturbances in several mechanisms related to the intestinal functions in the modulation of thyroid hormone metabolism: (a) the absorption of micronutrients (e.g., iodine, selenium, iron, zinc), which have a crucial role for thyroid function; (b) the storage of thyroid hormone through the competition of gut microorganisms with albumin for binding to iodothyronine; (c) the deiodination of thyroid hormones via the activity of deiodinases 1 and 3; and (d) the reabsorption of thyroid hormones in the enterohepatic circulation following the deconjugation of sulfate and glucuronide derivatives of thyroxine [84,86,90].

### 3.2. The Gut Microbiota and Autoimmune Thyroid Diseases: Epidemiological Evidence

In recent years, an increasing body of evidence has supported a decrease in α-diversity (community diversity and richness and species abundances) of the gut microbiota in subjects with AITD with respect to healthy controls [79,97,98,99]. The first meta-analysis to explore the association between gut microbiota and AITD included 15 studies and showed that Chao1, representing the index of the microflora richness, was increased in the HT group compared to controls, while it was decreased in GD [76]. The abundance of Lachnospiraceae, a family of bacteria belonging to the Firmicutes phylum and accounting for approximately 10% of the total gut microbiota, was higher both in subjects with HT and GD than in controls [100,101,102]. Importantly, within the Lachnospiraceae, many strains (e.g., *Roseburia homins*, *Roseburia intestinalis*, *Blautia producta*) capable of producing SCFAs have beneficial effects on the host health, while members of genera such as *Anaerostipes, Blautia,* and *Dorea* have been associated with intestinal and extra-intestinal diseases [103]. In addition, individuals with AITD displayed a significantly increased relative abundance of the genus *Bacteroides*, likely to contribute to the pathogenesis of inflammatory bowel disease [104], and *Bacteroides fragilis*, able to upregulate NLRP3, IL-18, IL-1β, and caspase-1 expression in enteric glial cells [100,105]. Fermentation of glucose and lactate by *Bacteroides* produces propionate, acetate, and succinate, which, since not being able to induce mucin synthesis unlike butyrate, may result in a reduction of intestinal tight junctions and increased permeability of the intestinal mucosa [79,106]. The meta-analysis by Gong et al. [106] reported a decreased proportion of beneficial bacteria such as *Lactobacillus* and *Bifidobacterium*, the main probiotic genera with health benefits for the host, including improvement of immune responses and intestinal impermeability and elimination of gastrointestinal microbial pathogens [107,108]. In particular, *Lactobacillus* may exert immune modulatory effects and suppress Th17 cell-mediated secretion of proinflammatory cytokine IL-17 through downregulation of IL-23 and TGF-β1 expression [109]. On the other hand, increased *Lactobacillus* levels were detected in patients with GD and were also significantly correlated with TSHRAb and the concomitant presence of TPOAb [79,110]. This finding supports data that indicate certain strains of *Lactobacillus* as being potentially harmful in susceptible individuals via mechanisms involving the synthesis of proinflammatory mediators, including ROS, cytokines, and participation in signaling cascades, such as the NF-kB and TLR2 pathways [111,112,113].

Interestingly, two case–control studies showed that HT patients were characterized by decreased abundance of *Prevotella* species (belonging to the phylum Bacteroidetes) [97,114], in turn being correlated with diets rich in carbohydrates and fibers, and whose prevalence in the gut is particularly relevant in the microbiome of non-Western cohorts [115]. While *Prevotella* is recognized as a producer of anti-inflammatory metabolites, resulting in a reduced Th17/Treg ratio, other studies have suggested that certain strains may exhibit pathobiontic properties in altering the composition and function of the microbial ecosystem and turning the intestinal immune system towards systemic autoimmunity by inducing the production of Th17 cells and reducing the IL-18 production [115,116,117,118].

Based on a recent systematic review and meta-analysis, including 16 studies for approximately 750 subjects with AITD and 488 controls, patients with both HT and GD displayed significant differences in the intestinal microbiota in terms of diversity and composition when compared to healthy individuals [12]. In GD, both richness (ACE and Chao1 indices) and diversity (Simpson and Shannon indices) presented lower scores than those occurring among controls, while significantly higher mean values were observed for all indices in HT, except for the Simpson index [12]. It is widely accepted that the Firmicutes/Bacteroidetes (F/B) ratio has a relevant influence on maintaining normal intestinal homeostasis, and variations in this score are related to dysbiosis [119]. Sawicka-Gutaj and co-authors [12] found that the abundance of Bacteroidetes was more pronounced in AITD patients, while that of Firmicutes remained unchanged, unlike Gong et al. [100], thus resulting in a lower F/B ratio than occurring among healthy participants. The authors also observed higher variability in the general abundance in GD than in HT, with respect to healthy controls, with a trend towards greater abundance of Bacteroidetes and Actinobacteria and of *Prevotella* and *Bifidobacterium* at the phylum and genus levels, respectively [12]. Furthermore, considering thyroid parameters, the most significant correlations were noted for TPOAb [12]. At the phylum level, Bacteroidetes negatively correlated with TSH and positively correlated with TPOAb and TSHRAb levels, while Synergistetes, whose reduced abundance appears to be associated with ulcerative colitis [120], showed inconsistent relationships with thyroid parameters [12]. Within Firmicutes, discrepancies were observed between studies: significantly negative correlations were reported between the relative abundance of *Vellonella* and *Streptococcus* and TSH, TPOAb, and TSHRAb [12] and between *Bacteroides*, *Faecalibacterium,* and *Coprococcus* and TPOAb or TSHRAb [100] in contrast to Sawicka-Gutaj et al. [12], who found conflicting results in correlations regarding *Bacteroides*, *Faecalibacterium,* and *Phascolarctobacterium*. *Faecalibacterium prausnitzii* is key in energy production for colonocytes through prebiotic fermentation, in significantly reducing the secretion of proinflammatory cytokines such as IL-12 and IFN-γ, and in inducing secretion of the anti-inflammatory cytokine IL-10; additionally, its levels were negatively correlated with inflammatory bowel disease and colorectal cancer [121]. *Phascolarctobacterium faceium*, which colonizes the gastrointestinal tract during early life up and reaches high levels in adulthood, is a massive producer of acetate/propionate from succinate, determining positive effects on the host [122].

In summary, while the results described made it impossible to classify a “good” or “bad” gut microbiota in absolute terms, due to the complex underlying mechanisms regulating the relationships of the gut microbiota with the host, there are signals of a positive association between decreased diversity and abundance indices of the microbial communities and the onset of AITD, with variations in the microbiota most significantly associated with TPOAb levels. Nonetheless, most of the published research was performed in Asia and did not always investigate the effects of internal (thyroid hormone and thyroid autoantibody levels, change of metabolism caused by AITD) and external factors (oral microbiome, diseases, dietary habits, drug use, seasonal variations) in this association, and therefore, they should be explored in future studies possibly involving various populations from different geographical regions.

### 3.3. The Gut Microbiota and Celiac Disease: The Underlying Mechanisms

Currently, especially in wealthier countries, heterogeneous clinical manifestations of CeD are present, from typical gastroenterological symptoms to a large number of extra-intestinal disorders [123,124]. In fact, in recent decades, the availability of highly sensitive and specific serological tests has made it possible to identify unsuspected cases of CeD presenting silent or clinical subtypes of the disease [125]. While it has been widely known that CeD is triggered by the ingestion of gluten in genetically predisposed individuals carrying the human leukocyte antigen (HLA) DQ2 or DQ8, other environmental factors, including the type of delivery and milk-feeding, antibiotic use, and intestinal infections, are hypothesized to represent additional elements involved in the pathogenesis of CeD [71,126]. Gluten is a very complex compound composed of two types of proteins: glutenin and gliadin [127]. Gliadin, rich in prolines and glutamines, which confer high resistance to gastric, pancreatic, and intestinal proteolytic digestion in the gastrointestinal tract, promotes adverse immune reactions, including mucosal inflammation, small intestinal villous atrophy, and increased gut permeability in CeD [127]. Indeed, in celiac patients, immune responses to gliadin are known to stimulate an inflammatory response mediated by both innate and adaptive immunity [68,126,128,129]:

1. The bond of gliadin peptides to the G-protein-coupled receptor CXCR3 on enterocytes leads to the secretion of zonulin, which, in turn, is responsible for the disruption of tight junctions and increased epithelial permeability. Of note, although gluten is capable of triggering zonulin release in both CeD and healthy individuals, the amount and duration of zonulin release are substantially higher in the CeD group;
2. The lectin wheat germ agglutinin, crossing the intestinal barrier, binds to the glycocalyx of human cells, enhancing intestinal permeability and inducing inflammatory responses by immune cells;
3. After translocation into the lamina propria, gluten-derived peptides are deamidated by intestinal tissue transglutaminase (tTG) into negatively charged glutamic acid residues. Such immunogenic molecules trigger the humoral immune response by activating B cells to release antibodies against gliadin and tTG and by promoting the production of pro-inflammatory cytokines (e.g., TNF-α, IFN-γ);
4. Immunogenic epitopes also stimulate an innate immune response in the intestinal epithelium through enhanced expression of IL-15 by enterocytes, in turn causing activation of DCs and intraepithelial lymphocytes, with the latter expressing the activating receptor NK-G2D, a natural killer cell marker, resulting in damage to intestinal tissue;
5. Following epithelial barrier disruption due to gliadin-mediated zonulin release, increased expression of IL-8, a key mediator in the innate immune response attracting and activating neutrophils in inflammatory regions, occurs in the epithelium and macrophages;
6. Within the framework of the adaptive immune response, the interaction with major histocompatibility complex (MHC) class II HLA-DQ2/8 located on antigen-presenting cells (APCs: DCs, macrophages, B cells, enterocytes) leads to the presentation of epitopes to CD4+ T cells that, by means of the secondary production of pro-inflammatory cytokines, like TNF-α and IFN-γ, generate a vicious cycle characterized by enhanced intestinal permeability and mucosal damage. This mechanism also involves a Th1-driven response to gliadin and an increase in Th17 cytokines, which ultimately results in a breakdown of tolerance and the development of chronic inflammatory conditions.

Importantly, the presence of one of HLA DQ2 or DQ8, which represents 30–40% of the genetic variance of CeD in Western countries, although necessary, is not sufficient to develop the disease, whereas the non-MHC susceptibility loci account for about 15% of the disease risk, overall [130,131].

### 3.4. The Gut Microbiota and Celiac Disease: Epidemiological Evidence

As we discussed in the previous sections, the gut microbiota plays a substantial role in regulating the development, homeostasis, and function of both innate and adaptive immune cells [132]. The possible contribution of environmental factors in the rising incidence of CeD and their close association with the microbiota composition shed light on the involvement of the intestinal microbiota in the pathogenesis of this disease [71,132]. A number of cross-sectional studies evaluating fecal and duodenal microbiota associated with CeD reported a decreased abundance of beneficial species (*Lactobacillus* and *Bifidobacterium*) and an increase in those potentially pathogenic (*Bacteroides*, *Clostridium*, *Staphylococcus*, *Escherichia coli*) in celiac patients compared to healthy individuals, suggesting that the higher amount of Gram-negative and potentially pro-inflammatory bacteria in the intestine of subjects with CeD is potentially related to the symptomatic presentation of the condition [133,134,135,136]. Conversely, other studies found no statistically significant differences in the composition of bacterial communities in the upper small intestine between untreated CeD patients and non-CeD controls [137,138]. These differences may be sustained by individual microbial profiles that can affect the results, especially when in the presence of studies based on small sample sizes, as well as by the likelihood of microbial alterations in symptomatic patients who underwent gastrointestinal endoscopy [71]. Although CeD patients following a gluten-free diet (GFD) appeared to restore α-diversity and exhibited a nearly non-dysbiotic microbiota, their β-diversity parameters were significantly different from those of healthy controls [131]. Indeed, they were characterized by a reduced abundance of three taxa belonging to the Lachnospiraceae family (*Coprococcus eutactus*, *Coprococcus catus*, *Blautia*), which are among the main producers of SCFAs, and *Bifidobacterium* species, but increased levels of *Bacteroides* genus, as observed for microbial composition in untreated CeD individuals [131]. Such findings were confirmed by a recent systematic review of 13 studies amounting to a total of 212 celiac patients subjected to a GFD in comparison to 174 healthy controls and 176 celiac untreated patients, which ultimately showed the lack of a full restoration of commensal microorganism abundance in patients treated with a GFD [139]. The authors observed that *Bifidobacterium* strains were less abundant in celiac patients on a GFD compared to the control groups [115]. This species can increase the intestinal defenses, protecting the host against infection by *Escherichia coli* through the production of acetate [140], directly counteracting the toxic effects of gliadin through the attenuation of CD4+ T-cell-mediated immune response [141,142], suppressing the pro-inflammatory cytokine pattern characteristic of CeD and supporting IL-10 production [143]. In contrast, the increased levels of *Bacteroides* in the treated CeD group could be associated with beneficial effects since they are capable of protecting the host from pathogens and providing nutrients to other intestinal commensal microorganisms [129,144].

So far, a few prospective studies have evaluated the association between gut microbiota and CeD. Within the PROFICEL study conducted in Spain, infants were grouped for factors capable of influencing the gut microbial colonization process, including age, HLA-DQ status, and type of milk-feeding, and fecal microbiota was analyzed at 7 days and at 1 and 4 months of age [145]. In the first report assessing the effects of both milk-feeding type and HLA-DQ genotype on the gut microbial composition in healthy full-term infants with a family history of CeD, breast-feeding promoted the colonization of *Clostridium leptum* group and of certain *Bifidobacterium* species, while that of the *Bifidobacterium lactis*, *Escherichia coli*, and *Bacteroides fragilis* group and *Clostridium coccoides–Escherichia rectale* group was favored by formula-feeding [145]. Subsequent research reported a higher prevalence of some pathogenic bacteria (*Clostridium perfringens* and *Clostridium difficile*) in formula-fed infants compared to breast-fed infants, while *Escherichia coli* was specifically associated with the HLA-DQ2 genotype, probably due to the reduced abundance of *Bifidobacterium*, in agreement with previous findings [145,146] and indicating that factors such as formula-feeding and the HLA-DQ2 genotype differently affect the presence of pathogenic bacteria in the intestinal microbiota in early life [147]. Of note, DQ2+/DQ8+ infants featuring delayed introduction of gluten into the diet from 4–6 months to 12 months of age showed a positive effect on prolonging gluten, as well as lower incidence of CeD autoimmunity, with an overall relative absence of the phylum Bacteroidetes and a concomitant high abundance of Firmicutes, with a microbiota quite different than that of adults even at 2 years of age [148]. This finding was supported by a Finnish longitudinal study reporting no significant differences in the fecal microbial diversity or composition between infants who subsequently would have developed CeD and those who did not at 9 and 12 months of age, suggesting that early fecal microbiota composition would not have been associated with the CeD pathogenesis [149]. Furthermore, no significant differences in the composition of the fecal microbiota were reported between breastfed and non-breastfed infants during gluten introduction, both supporting the current lack of evidence about the potential protective role of breast-feeding against CeD at the time of gluten introduction and its association with the intestinal microbiota [149,150,151].

In summary, if celiac patients following a GFD have a microbial composition comparable to that of healthy individuals, they appear not to completely restore commensal microbial abundance. However, the cross-sectional design of studies reviewed does not allow for the collection of data on dietary habits over time and, in most investigations, information on antibiotic and probiotic therapies is lacking, which may influence the composition and functioning of the gut microbiota, as occurring with serological and genetic profiles of patients [139]. Data from prospective studies are sparse and conflicting; however, some evidence supports that the host genotype can influence the intestinal colonization of infants, although the lack of prolonged follow-ups does not allow the definite establishment of an association between these microbiota variations and subsequent development of CeD.

## 4. The Bidirectional Relationship between Autoimmune Thyroid Diseases and Celiac Disease

In AITD, the two main clinical presentations, HT and GT, occur due to a dysregulation of the immune system, resulting in an immune attack on the thyroid [5]. If in HT, the lymphocytic infiltration of the gland, together with the secretion of TPOAb and TGAb, induces follicular cell destruction, necrosis, and apoptosis, resulting in fibrosis (and potentially hypothyroidism), the major features in GD include a predominant humoral response with persistent production of TSHRAb, which can cause goiter, hyperthyroidism, ophthalmopathy, and dermopathy [152]. While the association between AITD and other organ-specific or systemic autoimmune disorders, including CeD, is largely known, the pathogenetic processes linking AITD and CeD have yet to be fully established [5,153]. The prevalence of CeD in AITD patients was initially estimated to range between 2 and 7.8% [154]; then, a meta-analysis based on 6024 AITD patients and a total of 27 studies reported a prevalence of biopsy-confirmed CeD of 1.6%, doubling the prevalence in the general population, with higher frequency among children than among adults (6.2% vs. 2.7%) [23]. Subsequently, an Indian tertiary hospital-based study screening 280 consecutive AITD patients for tTG antibodies estimated a prevalence of celiac autoimmunity of 8.6%, although this result could not be confirmed by tissue diagnosis in all patients [155]. According to a prospective work dealing with 66 Turkish children with AITD tested for tTG IgA, CeD had a frequency of 3%, suggesting a tight relationship between these two autoimmune disorders [156].

Importantly, the interaction between CeD and AITD is bidirectional and, as shown in a landmark study by Ventura et al. [157], the prevalence of autoimmune disorders in adolescents and young adults with CeD is significantly higher than in the general population and is related to the increased age at diagnosis and, consequently, the duration of exposure to gluten. A meta-analysis including 13 studies for a total of 15,629 CeD cases and 79,342 controls showed an increased prevalence of thyroid disease among celiac patients compared with that in the control groups (Odds Ratio − OR = 3.08, *p* < 0.001) without noticing differences between the gluten-treated and untreated groups [19]. Notably, patients with CeD had more than a four-fold risk of developing euthyroid AITD than patients without CeD [19]. A subsequent large, cross-sectional, population-based study on 2,001,353 Jewish Israeli adolescents, including 7145 subjects with CeD and 1,580,896 controls, confirmed that autoimmune diseases were significantly more frequent in celiac subjects, with an increased risk of 1.8 times for thyroid diseases [158]. In a case–control study conducted in Sardinia, an Italian region characterized by an extremely high prevalence of autoimmune disorders, including AITD, CeD, and type 1 diabetes, and based on 623 celiac patients and 7866 controls, the authors reported an overall frequency of thyroid diseases twice as high in celiac patients (26.0%) than in controls (12.9%) [22]. In particular, the AITD risk was increased in patients with CeD (15.4%) compared to patients without CeD (7.5%), and the difference was reported as statistically significant in both euthyroidism and hypothyroidism conditions [22]. AITD remained strongly associated with CeD (OR = 2.387, *p* < 0.001) in the adjusted model of multivariate analysis, and significant associations were also shown with female sex (OR = 5.855, *p* < 0.001) and age (OR = 1.012, *p* < 0.001) [22]. Previously, within a retrospective single-center case–control study involving 255 CeD patients treated with GFD and 250 controls, the same authors demonstrated that HT was the most prevalent autoimmune disease among celiac individuals, with 2.5-fold increased risk (*p* < 0.0001) and no significant difference by sex [124]. Moreover, in agreement with Ventura et al. [155], disease duration was a significant predictor of additional autoimmune disorders, indicating that the increased risk of autoimmunity might be mainly linked to common genetic features in these subjects [124]. Of interest, first-degree relatives of patients with CeD had a three-fold higher prevalence of AITD (17.5% vs. 5.03%, *p* = 0.0013) and associated thyroid dysfunction (11.8% vs. 3.5%, *p* = 0.010) than healthy controls, although the lack of a baseline biochemical assessment to select healthy controls represents one of the major limitations of this research [159].

Overall, although the major findings come primarily from cross-sectional or retrospective case–control studies conducted in Western countries, current evidence supports the possible, also relatively frequent, coexistence of AITD and CeD, suggesting the need to routinely monitor thyroid function in celiac individuals at presentation and follow-up, as well as to screen AITD patients for CeD autoantibodies, even if asymptomatic, in order to also provide data about the chronological development of these conditions in individual subjects. Nonetheless, multicenter prospective studies involving a larger number of patients should be performed to produce more reliable prevalence estimates and thus confirm the close relationship between AITD and CeD, whereas the effects of GFD on the occurrence of AITD in celiac patients should be further investigated.

### 4.1. The Mechanism Underlying the Coexistence of Autoimmune Thyroid Disease and Celiac Disease

**Figure 2:**
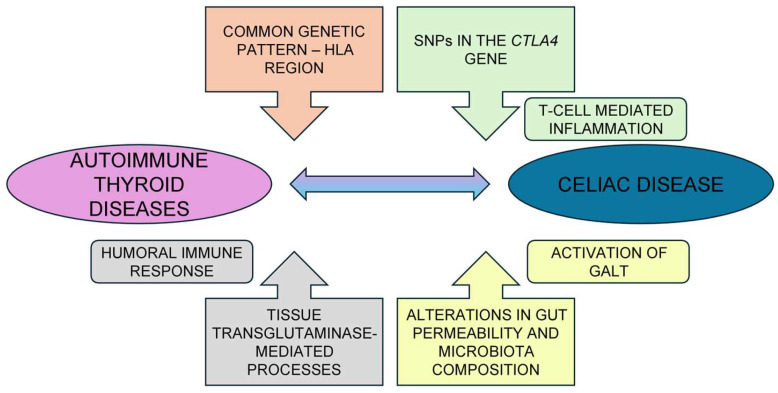
Summary of the common processes possibly related to the coexistence of autoimmune thyroid diseases and celiac disease. Abbreviations: *CTLA4*: cytotoxic T-lymphocyte-associated antigen 4; GALT: gut-associated lymphoid tissue; HLA: human leukocyte antigen SNPs: single-nucleotide polymorphisms.

The co-existence of CeD with other autoimmune disorders, including AITD, and vice versa, probably implies a potential role for gluten peptides, which are the known etiological agent of CeD but could also be involved, directly or indirectly, in the onset of AITD, whose etiopathogenesis is deemed multifactorial, albeit not fully clarified [159]. Some direct and indirect mechanisms have therefore been hypothesized to try to explain the concomitance of CeD with AITD (Figure 2):

1. The association between CeD and AITD might be supported by the presence of a common genetic background consisting of multiple genetic loci involved in the development of autoimmune disorders [124,158]. The HLA region, containing more than 200 genes grouped in three main classes (I; II, which includes the heterodimeric HLA-DR, DP, and DQ genes; and III) and presenting both extremely high polymorphism and codominance inheritance (proteins resulting from the expression of both alleles), became the first candidate genetic region to be fully investigated for association with autoimmune diseases [136]. In Section 3.3, we have already mentioned HLA-DR3-DQ2 and DR4-DQ8 as the most relevant and best-characterized genetic determinants in CeD. Hence, the presence of any of the DQ2.5, DQ8, or DQ2.2 heterodimers increases the risk of developing CeD; in particular, the HLA-DQ2.5 heterodimer, one variant of the DQ2 molecule encoded by the DQB1*02 and DQA1*05 alleles, represents the most permissive heterodimer for CeD (encoded by approximately 90% of celiac patients). On the other hand, most subjects with genetic susceptibility fail to experience the disease. Regarding AITD, both HT and GD have multiple common etiological and pathophysiological factors, including HLA-predisposition [137]. At-risk haplotypes for GD include DQA1*0501 (linked to DR3 and to DR5) and DQB1*0302 (linked to DR4), while at-risk haplotypes for HT include HLA DQA1*0301 (linked to DR4), DQB1*0301 (linked to DR5) and DQB1*0201 (linked to DR3) [160,161,162,163,164,165].

2. In addition to the HLA region, several studies have also explored the involvement of functional variations of the cytotoxic T-lymphocyte-associated antigen 4 (CTLA4) gene in a number of autoimmune disorders. *CTLA4* may play a crucial role in the negative regulation of T-cell-mediated inflammatory responses and contains several single-nucleotide polymorphisms in the 3′ region, strongly associated with the development of type 1 diabetes and AITD [166,167].

3. As previously reported (Section 3.3), the enzyme tTG is in charge of deamidation and the consequent immunogenicity of gliadin, which, in the deamidated form, may interact with HLA DQ2 or DQ8 on APC. Interestingly, tTG has also been detected at high levels in other tissues, including the thyroid gland. In the thyroid, it can be detected both in the cytosol and in the follicle lumen, where it acts in the cross-linking of thyroglobulin, the macromolecular precursor of thyroid hormones and the most abundant protein in the gland, representing up to 75% of the total protein content [168]. Nayer et al. [169] demonstrated the bond of serum anti-tTG antibodies to tTG located in thyroid tissue and the positive correlation between tTG IgA and TPOAb titers in patients with active CeD, thus speculating a role for anti-tTG antibodies as a contributing factor in the development of thyroid autoimmunity.

4. In Section 3, we described the key role of the gut microbiota in the normal function of the immune system, GALT development, and immune tolerance to autoantigens in the intestinal mucosa. Conversely, a leaky gut and alterations in the composition of the intestinal microbiota might lead to the transfer of antigens into the bloodstream, where autoreactive autoimmune cells are generated at the level of lymphatic connection of peripheral organs or, alternatively, to the direct migration of autoreactive intestinal immune cells toward target organs, where systemic inflammation and autoimmunity are activated [25,170,171].

### 4.2. Immunomodulatory Role of Vitamin D

**Figure 3:**
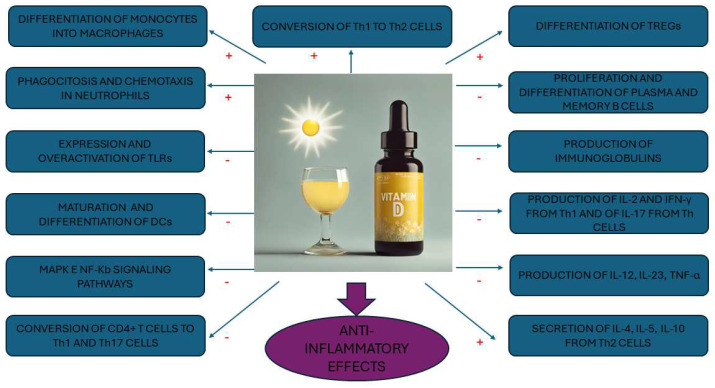
Effects of vitamin D on the immune system. The symbols + and − in the figure indicate inducing and inhibitory effects, respectively. Abbreviations: DCs: dendritic cells; IFN-γ: interferon gamma; IL: interleukin; MAPK: mitogen-activated protein kinase; NF-κB: nuclear factor kappa-light-chain-enhancer of activated B cells; Th cells: T helper cells; TLRs: Toll-like receptors; TNF-α: tumor necrosis factors alpha; Tregs: T regulatory cells.

As mentioned in Section 2, along with the classical hormonal effects reported on maintaining mineral homeostasis and skeletal health, vitamin D also exerts immunomodulatory actions thanks to the expression of VDR and 1-α-hydroxylase in most immune cells [172]. A series of mechanisms may support the immunomodulatory role of vitamin D and its potential involvement in AITD (reviewed in [28,172]) (Figure 3):

1,25(OH)_2_D3 has a major influence on the innate immune response, considered the first line of defense against infections. In particular, it is key to the promotion of the differentiation of monocytes into macrophages, to induce phagocytosis and chemotaxis in neutrophils, and to suppress inflammation by inhibiting expression and overactivation of TLRs on the surface of monocytes.

1,25(OH)_2_D3 inhibits the maturation and differentiation of DCs, a type of APC capable of initiating adaptive immune responses and modulating immune tolerance. This action leads to a reduction in antigen uptake and presentation and to the activation of naïve T cells, resulting in suppression of autoimmunity. Vitamin D also reduces the production of cytokines and chemokines (IL-12, IL-23, TNF-α, IFN-γ) involved in the synthesis of Th1 and Th17 cells by inhibiting the activation of p38 MAPK and NF-κB signaling pathways in DCs and stimulates the production of anti-inflammatory IL-10.

As part of the adaptive immune response, 1,25(OH)_2_D3 has regulatory effects on both humoral and cell-mediated immune responses through a direct action on the maturation, differentiation, and apoptosis of both T and B lymphocytes, as well as on the regulation of their activities. Indeed, vitamin D can affect the distribution of Th1, Th2, and Th17 cells, inhibiting the conversion of CD4+ T cells to Th1 cells and Th17 cells and promoting the conversion of Th1 to Th2 cells. It also suppresses inflammation by inhibiting the production of IL-2 and IFN-γ from Th1 cells and of IL-17 from Th17 cells and fostering the secretion of IL-4, IL-5, and IL-10 from Th2 cells. In addition, vitamin D induces the differentiation of Tregs and their secretion of IL-10, thereby restoring the Th17/Treg imbalance (a hallmark of inflammation) and counteracting autoimmune diseases, including AITD. As for B cells, 1,25(OH)_2_D3 may affect their function directly, suppressing the proliferation and differentiation of plasma cells and memory B cells and decreasing the production of immunoglobulins (e.g., IgA, IgE) indirectly by altering CD4+ T-cell responses or, alternatively, inhibiting cytokine secretion.

### 4.3. The Association between Vitamin D and Autoimmune Thyroid Diseases

A growing body of evidence suggests a relationship between vitamin D and the risk of HT, with the results remaining somewhat conflicting. The meta-analysis by Wang et al. [29], including 20 studies published by the end of 2014 for a total of 1782 AITD cases and 1821 controls, reported that AITD patients had significantly lower levels of 25(OH)D3 than controls with more likely to present deficiency in vitamin D compared to healthy individuals. The same findings were also observed exclusively for HT or GD [29]. Nonetheless, the authors admitted that a variety of factors might have caused the heterogeneity of the results, including the variability of assays and cut-off values to determine vitamin deficiency, methods of AITD diagnosis, studies based on case–control design, which are unable to establish a causal relationship [29]. The meta-analysis by Xu et al. [173], aimed at evaluating the association between vitamin D levels and GD, included 27 studies published by the end of April 2015, most of them from China, summing up to 1770 cases and 1946 controls. The authors found that patients with GD reported a higher risk of vitamin D deficiency than controls (OR = 2.24, *p* < 0.001), despite a high degree of heterogeneity, mainly due to vitamin D assay methods [156]. The meta-analysis by Štefanić and co-workers [174], including 25 studies published up to February 2018 for a total of 2695 subjects with HT and 2263 controls, confirmed lower serum 25(OH)D3 concentration in HT compared to healthy controls. In particular, HT showed an OR of 3.21 (*p* < 0.001) for 25(OH)D3 deficiency (cut-off 20 ng/mL) with respect to healthy controls, although substantial heterogeneity was observed between studies [174]. It is important to note that the studies included in the meta-analysis did not cover many world areas (e.g., Americas, Australia, Africa), low-income countries, equatorial or high-latitude areas, or participants of Black ethnicity. Furthermore, individual information on sun exposure, skin phototype, and dietary vitamin D intake was not available from the studies, while the measurements of circulating 25(OH)D3 concentration could not always be comparable due to the absence of standardized test methods [174]. A systematic review and meta-analysis searching for observational studies about the association between vitamin D levels and thyroid disorders in the adult population and published up to March 2021 showed significantly lower serum levels of 25(OH)D3 in patients with AITD than observed in healthy counterparts, taking into account thirteen datasets for a total of 12,916 participants (1886 individuals with AITD and 11,030 controls) [175]. A significantly negative association was also observed among HT patients compared to healthy individuals, whereas serum vitamin D did not correlate with GD, except for subjects aged 40 or older [175]. Finally, a recent systematic review including 11 studies published between 2011 and 2021 and evaluating a total of 1952 AITD cases supported previous results, demonstrating that in most research, patients with HT and GD had a greater prevalence of vitamin D deficiency or low serum 25(OH)D levels compared with healthy subjects [176].

Overall, various factors may be in charge of the significant heterogeneity observed among the studies reviewed. In addition to the inter-assay differences already mentioned, other variables, including seasonal variations and BMI, should be considered as confounders [175]. Importantly, VDR not only modulates the biological effects of vitamin D, but certain polymorphisms in the gene receptor may be able to affect vitamin D function [177]. A meta-analysis evaluating the association between *VDR* gene polymorphisms and AITD risk, including eight studies (updated in August 2012), found that BsmI or TaqI polymorphisms were significantly associated with increased risk of AITD in contrast to ApaI or FokI polymorphisms, indicating that individual genetic susceptibility might influence the onset of AITD [178]. Conversely, a subsequent meta-analysis dealing with eleven case–control studies (updated in September 2016) showed that only the FokI polymorphism was significantly and positively associated with the risk of HT in the global population, as well as in Asians, yet not in Caucasians [177]. Other research reported the positive association of two single-nucleotide polymorphisms in VDR with GD, although no significant difference in mean vitamin D level was found between genotypes, suggesting that VDR may influence GD risk through other mechanisms beyond a reduced serum concentration of vitamin D [179].

Along with a complex interaction of immune cells and cytokines with thyrocytes, different T-cell subpopulations have been recognized to play a direct or indirect role in the pathogenesis of AITD [180]. Indeed, the main traits in the development of HT include the Th1/Th2 cell imbalance and the enhancement of Th1 and Th17 cell activities [181,182,183], which, by triggering various mechanisms involving both humoral and adaptive immune responses, lead to an uncoupling of the interplay between different immune components up to the destruction of the thyroid tissue (reviewed in [180]). Unlike HT, characterized by a Th1 pattern of immune response typical of cellular immunity, in GD, there is instead a predominance of Th2 cytokines, a hallmark of humoral immune response [184]. Cholecalciferol supplementation can modify the balance of CD4+ T-cell subsets, resulting in a significant decrease in Th17/Th1 ratio in female patients with HT compared to the placebo group, in line with the beneficial immunological effects described in Section 4.2 [185]. Furthermore, vitamin D can downregulate HLA class II gene expression in the thyroid, in turn leading to reduced antigen presentation and T-cell activation, thereby inhibiting inflammatory cytokine synthesis [28,186]. On the other hand, inconsistent results were seen about the association between vitamin D status and TPOAb in HT compared to the control group, with negative [187,188] as well as positive correlations [189] and lack of association [190,191]. A recent systematic review and meta-analysis by Wang et al. [192], including six randomized controlled trials for a total of 344 patients with HT, reported that vitamin D supplementation significantly reduced TPOAb titers at six months but not at three months, as well as lowered TGAb levels. The recent randomized, double-blind, placebo-controlled clinical trial by Bhakat et al. [193] supported the correlation between the treatment with cholecalciferol weekly for 8 weeks and a significant decrease in TPOAb levels. Interestingly, in a study conducted in China on 54 children with GD and 36 with HT [194], serum 25(OH)D3 concentration was negatively correlated with serum IL-21 levels in both groups of patients. Together with its receptor, IL-21 is upregulated in AITD and, playing a role in innate and acquired immune responses, could be involved in the pathogenesis of the disease [194]. In contrast, in a Brazilian study assessing vitamin D status in relation to cytokines produced by Th1 (IL-2, IFN-γ, TNF-α), Th2 (IL-4, IL-5), and Th17 (IL-17) cells in 88 patients with HT and 71 euthyroid healthy subjects, the authors observed a positive correlation between vitamin D and IL-17, TNF-α, and IL-5 in HT patients only, which might be explained by the control of cytotoxicity resulting from long-time treatment of HT based on levothyroxine replacement therapy [195]. Importantly, no significant difference was observed in vitamin D concentration between the two groups, while the positive correlation between vitamin D and free thyroxine (fT4) in patients with HT might reveal the beneficial effect of levothyroxine in maintaining vitamin D in a status of sufficiency [195].

In analyzing the relationship between vitamin D and risk of GD, Zhang et al. [196] reported a significantly lower serum level of 25(OH)D3 from Chinese TSHRAb-positive GD patients compared to healthy controls or TSHRAb-negative patients, suggesting a putative role of vitamin D status in increasing thyroid autoimmunity in GD patients. No significant association was otherwise observed between 25(OH)D3 level and fT4, free triiodothyronine (fT3), TSH, TPOAb, or TGAb [196]. Within a large Swedish study involving 292 subjects newly diagnosed with GD and 2305 controls, the authors confirmed the absence of a significant association of vitamin D levels at diagnosis with fT4 and fT3, but unlike Zhang et al. [196], they found no correlation of 25(OH)D3 levels with TSHRAb, with Graves’ ophthalmopathy at diagnosis, nor with recurrence after discontinuation of anti-thyroid drugs (ATD) treatment [179]. Ahn et al. [197] reported that in 143 Chinese patients diagnosed with CD and who discontinued ATD, serum 25(OH)D3 levels at the time of ATD discontinuation did not correlate with either TSHRAb or TSH-binding inhibitory immunoglobulins (TBII), although associated with a higher incidence of GD recurrence. A prospective cohort study conducted in China, which recruited 210 subjects with GD and vitamin D deficiency, showed no significant difference in the rate of disease recurrence between the subjects who received cholecalciferol (29% of the total) one year after suspension of ATD and those who did not (71% of the total), even if the latter group reported an earlier relapse (*p* = 0.016) [186]. Nonetheless, both selection bias and the difficulty in regularly monitoring medication compliance may have affected the results. Moreover, a weak negative correlation was observed between vitamin D levels and TBII titers, and both parameters represented significant factors for AITD relapse [186]. Serum vitamin D levels were also significantly lower in Japanese female GD patients without remission (n = 18) compared to those with active disease (n = 36), although the cross-sectional design and the limited number of subjects involved did not allow to define the relationship between vitamin D and the pathogenesis or prognosis of GD [198].

In summary, an increasing amount of evidence suggests the association between vitamin D deficiency or insufficiency and AITD risk, which is also supported by the immunomodulatory role of vitamin D shown in experimental and mechanistic studies, although prospective studies enrolling populations from different world areas and evaluating additional confounders are currently missing. Vitamin D supplementation, if shown to have some effects in HT reducing autoimmunity, does not appear to substantially prevent GD recurrence, nor do consistent findings indicate a correlation between serum vitamin D levels and thyroid parameters or anti-thyroid antibodies in GD patients (Table 2). Further clinical trials enrolling various cohorts and analyzing the genetic expression of VDR would be desirable to also explore the effects of higher doses of vitamin D supplementation.

### 4.4. Vitamin D and the Effects of Vitamin D on Intestinal Host–Microbiome Interactions

Mounting evidence has highlighted the potential of vitamin D to influence the composition of the gastrointestinal microbiome [199]. Within a systematic review, including 10 mouse and 14 human studies, a total of 22 studies out of the 24 reviewed reported an association between vitamin D β-diversity, but not α-diversity, of the gut microbiome [200]. Notably, the *VDR* gene was associated with β-diversity in both humans and mice, although most human studies were observational and adjusted for a limited number of confounding factors [200].

Some indirect mechanisms may explain the relationship between vitamin D and the microbiome as follows.

The complex calcitriol-VDR directly induces transcription of genes encoding antimicrobial peptides (AMPs) (defensins and cathelicidins constitute the two major classes) in intestinal secretory epithelial cells, i.e., Paneth cells, and in immune cells in the gastrointestinal tract. These molecules represent conserved effectors of the innate immune system within the intestine with critical roles in maintaining tolerance to commensal microbiota and protecting against enteric infections [201,202]. Consequently, AMPs can shape the composition of the gut microbiome in a bidirectional and dynamic process; therefore, species-specific AMP profiles are capable of preserving species-specific bacterial communities, while, on the other hand, through the production of SCFAs, the microbiota directly regulates AMP production and functioning [202].

Vitamin D is a powerful stimulator of NOD2, a cytosolic PRR, and its downstream pathways (NF-κB, MAPKs, and Caspase-1), which in turn stimulate host innate and adaptive immune responses. In the intestine, NOD2 is expressed by numerous cell types, both hematopoietic and non-hematopoietic cells (e.g., Paneth cells, stem cells, Goblet cells, enterocytes). Given the role of NOD2 in the control of microbial infection, its deficiency has been linked to dysregulation of microbiota composition and the onset of Crohn’s disease and inflammatory bowel disease [203,204,205]. Vitamin D/VDR signaling also downregulates both mRNA and protein levels of PRR transmembrane proteins TLR2 and TLR4, which results in decreased nuclear translocation of NF-κB, thereby, in turn, reducing inflammation [206,207].

Vitamin D/VDR signaling directly or indirectly influences the expression and function of cell junction proteins, including claudin-2, -5, -12, and -15 in intestinal epithelial cells, which are primarily responsible for regulating the integrity of the intestinal barrier and the passage of harmful micro-organisms to the lamina propria [208,209]. Increased intestinal permeability, which triggers inflammation and downstream effects on the gut microbiota, is one of the early events preceding the onset of autoimmune disorders, including CeD [208]. The relationship between vitamin D and the composition of the intestinal microbiota is bidirectional; therefore, supplementation with probiotics, by improving VDR expression, might inhibit pathogenic bacterial invasion and intestinal inflammation [199,210].

### 4.5. Vitamin D and Celiac Disease

In previous sections, we reported that CeD generally occurs in genetically susceptible individuals who, in the presence of specific environmental factors (modality of infant feeding, type of delivery, antibiotic therapies, intestinal infections), manifest an immune response triggered by gluten intake. Interestingly, several studies assessing the risk of developing CeD in relationship to seasonal variability have shown that summer birth appears to be associated with an increased risk of later developing CeD due to both complementary feeding (gluten) in winter and seasonal fluctuations in vitamin D levels [208,211]. Furthermore, the association between CeD prevalence and geographic region can be possibly attributed to differences in the exposure to sunlight, which is generally lower at northern latitudes and predisposes to vitamin D deficiency, thus explaining the north–south gradient in the onset of CeD [212]. In evaluating the role of vitamin D status in the onset of CeD, the meta-analysis by Lu et al. [213], including 24 articles and 25 datasets containing 1137 CeD patients and 2613 controls, reported that celiac patients had decreased serum 25(OH)D3 levels, which were restored to normal values (i.e., comparable to healthy controls) after a GFD treatment. In particular, no significant differences were found in 25(OH)D3 levels between pediatric CeD and controls, but only between the adult groups (average 25(OH)D3 concentration 21.92 nmol/L lower in celiac patients than in controls), probably due to greater exposure to sunlight and greater frequent use of foods fortified with vitamin D in the first years of life [24,213]. Nonetheless, although the results of this meta-analysis indicate that vitamin D may play a crucial role in CeD pathogenesis, the cross-sectional design of the studies included is unable to define the directionality of this association [213]. A recent systematic review and meta-analysis, including 26 studies published in the years 2000–2023, amounting to a total of 3120 subjects (1495 CeD patients and 1607 control participants), showed that vitamin D levels in pediatric CeD patients were lower compared to healthy controls, and 25(OH)D3 deficiency was more prevalent in CeD patients (OR = 2.29, *p* < 0.0001) [214]. Gastrointestinal infections and rotavirus infections in children are strongly associated with a subsequent diagnosis of CeD [215,216], while vitamin D, as also widely discussed in this paper (Section 4.2), has protective effects against infections by regulating the activity of both innate and adaptive immune systems [214]. However, the limited number of the studies included, their high heterogeneity, and the lack of prospective studies and randomized controlled trials represent significant limitations of the meta-analysis by Sun et al. [214]. Of note, a case–control study nested within the Norwegian mother and child cohort study, in which 25(OH)D3 concentration was measured in maternal blood from mid-pregnancy, postpartum, and cord plasma of 416 children who developed CeD and 570 randomly selected controls, observed no significant difference in mean 25(OH)D3 between the two groups, even after adjusting for genetic risk markers [217]. On the other hand, in a cross-sectional study performed on 200 Saudi adolescent girls (range of age 13–19 years) with vitamin D deficiency, participants with serum 25(OH)D3 < 50 nmol/l had positive tTG IgA antibodies 37.2 times higher compared to those with higher vitamin D levels, suggesting the need for screening for CeD asymptomatic patients with severe-to-extreme vitamin D deficiency [218].

A lifetime GFD is the currently available and best-known treatment for CeD [219]. Indeed, GFD allows recovery of small intestinal histology in 94% of children [220] and 76% of adult patients within 2 years [221], supporting that treatment of patients with CeD should be started in early age, given the significantly greater probability of regression of gastrointestinal symptoms in younger individuals [219]. In patients with CeD, long-term mucosal damage of the small intestine and inflammation lead to malabsorption of nutrients, such as calcium and vitamin D, thereby increasing the risk of short height, delay of puberty, and osteoporosis [219,222]. GFD can improve symptoms of malabsorption (e.g., diarrhea, weight loss) and ameliorate bone mineralization in celiac patients [219,222]. Indeed, the aforementioned meta-analysis by Sun et al. [214] reported a significant increase in 25(OH)D3 levels following a GFD treatment [214]. In particular, one of the nine prospective studies included in this analysis and performed on newly diagnosed 60 pediatric celiac patients confirmed a significant increase (*p* < 0.001) in vitamin D levels regardless of the hormonal concentration at the onset of disease (insufficient, deficient, sufficient), as well as in bone mass density (BMD) and bone mass content after 6 months on a GFD [223].

Importantly, the GFD is poor not only in dietary fibers but also in micronutrients, mostly vitamin D, vitamin B12, and folate, as well as essential elements like iron, zinc, magnesium, and calcium [30]. As observed by Di Nardo et al. [224] within a systematic review, nutritional deficiencies in children can be exacerbated by GFD; therefore, celiac patients should be encouraged to use local naturally gluten-free foods, including pseudo-cereals (e.g., maize, millet, rice), green vegetables, legumes, fish, or, alternatively, gluten-free products enriched with vitamins and/or minerals. Consistently, although a Spanish cross-sectional study involving 64 celiac patients on a long-term GFD and 74 sex- and age-matched non-celiac controls did not reveal significant differences in vitamin D and other micronutrient (e.g., vitamin E, calcium, iron, zinc) intake between the two groups, it found a moderate deficiency of vitamin D (between 10 and 30 ng/L) in 34.4% of CeD patients, which suggests that patients with CeD should be accurately monitored to assess any nutritional deficiencies [225]. Indeed, a single-center retrospective chart review based on data from 130 Dutch pediatric individuals with CeD aged 0 to 18 years reported several nutritional deficiencies between 3 months and 10 years after starting a GFD; vitamin D deficiency was observed in 21.1% of measurements, which included persistent deficiencies after diagnosis and newly developed ones [226].

It is important to report that, in non-GFD conditions, while plasma levels of 25(OH)D3 are often low in celiac patients, the plasma 1,25(OH)_2_D3 levels can be frequently elevated in CeD, especially in newly diagnosed patients; thus, the presence of vitamin D deficiency in untreated patients is a rare event [227,228]. Elevated levels of 1,25(OH)_2_D3 are, in turn, related to high levels of PTH, which upregulates the conversion of 25(OH)D3 to 1,25(OH)_2_D3 and can induce bone resorption [227,229]. Therefore, if high levels of PTH are significantly and inversely correlated with bone mineral density (BMD) in adult patients with CeD [230], a strict GFD generally normalizes these alterations after at least 1 year of treatment [231,232,233]. Interestingly, an Italian study collecting data from 105 CeD patients for lab tests found that untreated CeD subjects had 22.0% lower serum 25(OH)D3 (*p* = 0.023), 42.5% higher serum PTH (*p* < 0.001) but without alterations in serum calcium and phosphorus, and 13.0% higher serum 1,25(OH)_2_D3 (*p* = 0.029) compared to subjects on a GFD [234]. These results supported the hypothesis that low serum 25(OH)D3 levels are likely to be attributable to an inhibitory effect of PTH secretion, which, in turn, is responsible for the higher serum 1,25(OH)_2_D3 [217]. Notably, vitamin D status was not associated with BMD, casting doubts about the effectiveness of vitamin supplementation in celiac patients, which was in line with the guidelines of the American College of Physicians for BMD and osteoporosis [234]. As reported by Verma and co-authors [223], patients with vitamin D deficiency (<12 ng/mL) who received 60,000 IU of vitamin D per week during the first 3 months of treatment together with GFD, although showing a significant increase in 25(OH)D3 levels, did not reach normal vitamin D values. Moreover, the putative role of vitamin D in increasing the risk for CeD cannot be ruled out. An American Internet-based survey carried out among parents with at least one biological child and aimed at determining which variables were best correlated with CeD in children first showed that vitamin D drops administered for longer than 3 months were significantly associated with an increased risk of CeD (OR = 1.749, *p* < 0.025) [235]. Conversely, a longitudinal observational study evaluating 6627 children recruited at six clinical research centers in the United States and Northern Europe, of whom 1136 developed CeD autoimmunity and 409 CeD, demonstrated that dietary supplementation during pregnancy, including vitamin D in 94% of mothers, did not modify the risk for the disease in the offspring [236].

Overall, while it is shown that vitamin D is involved in protecting the gastrointestinal tract by modulating the gut microbiota composition and the immune system, lower 25(OH)D3 levels have not always been observed in celiac patients. Additionally, the effectiveness of vitamin D supplementation in CeD treatment remains uncertain, even in light of treatment with a GFD that might normalize serum levels of both 25(OH)D3 and 1,25(OH)_2_D3 (Table 3). Thus, longitudinal studies with adjustment for additional relevant confounding factors (BMI, dietary habits) and randomized controlled studies with a large sample size and prolonged follow-up could help to clarify the consistency and directionality of these findings and to define the role of this nutrient in the pathogenesis of CeD.

## 5. Novel Strategies for Nutritional Supplementation Based on Vitamin D

As stated, among the major sources of vitamin D are edible substances, whose consumption is key to ensure the necessary supplementation of this important nutrient to rule out a possible contribution to the pathophysiology of HT, GD, and other possibly related conditions. In some particular cases, the supplementation of vitamin D can be questionable and could also generate some serious health concerns under the vitamin D intoxication umbrella effects, which, although basically impossible due to sunlight exposure and dietary intake, becomes a realistic effect of the excessive ingestion of vitamin D supplements [237,238]. However, despite the poor likelihood of its occurrence, vitamin D intoxication effects, such as hypercalcemia and/or hypercalciuria, could persist for long periods even after the removal of supplementation due to the lipophilic properties and storage of vitamin D in fat tissues [239]. Due to the consequences of severe hypercalcemia and hyperphosphatemia, potentially affecting gastrointestinal, renal, nervous, cardiovascular, and musculoskeletal systems, the argument should be considered very carefully when prescribing or managing vitamin D supplementations. In this regard, despite attempts made by important clinical institutes and regulatory agencies to suggest “universal” supplementation levels, the topic remains controversial, and personalized approaches should be conceived and carried out to maximize positive outcomes, decreasing the likelihood of collateral effects and maximizing the compliance of an individual to the prescribed diet or therapy.

### 5.1. Personalized Vitamin D Levels: The Role of Artificial Intelligence

Thanks to the progress of technology in various aspects of everyday life, Artificial Intelligence (AI) has become pivotal in the field of healthcare and well-being, especially when it comes to arguments running around the “p4 medicine”. Its invaluable support in detecting subtle relationships between variables within structured or non-structured datasets makes AI ideal for predicting disease outcomes and the responses to pharmacological or non-pharmacological treatments, even within the framework of a personalized perspective. In this regard, several works have been published in recent years that apply AI in clinical questions around the usage and eventual effects of vitamin D supplementation in various categories of diseases around the globe.

For example, feed-forward Artificial Neural Networks (ANN) with back-propagation as the training algorithm in 70% of data as the training set and 30% of data as the validation set have been employed to investigate the relationship between neuropsychological function and responsiveness to vitamin D supplementation in a cohort of over 600 individuals, finding a predictive ability for responsiveness to vitamin D supplementation with sensitivity and specificity between 0.60 and 0.70 and 0.66 and 0.70, respectively. Cognitive abilities (42.5%), basal vitamin D (21.3%), BMI (9.5%), and daytime sleepiness (8%) turned out to be the most predictive variables with respect to changes in serum vitamin D levels [240].

Later on, a Chinese group used AI and Machine Learning, in particular, to predict the relationships between vitamin D status and the occurrence of neurological deficits in a cohort of 200 patients affected by cerebral infarction, specifically using a Logistic Regression and XGBoost model, dividing training and test sets in a 7:3 ratio [241]. In the test set, the AUC of the Logistic Regression and XGBoost was 0.761 (95% CI: 0.640~0.882) and 0.786 (95% CI: 0.670~0.902), respectively, demonstrating a good prediction accuracy overall.

Even more specific is the prediction of vitamin D deficiency in a cohort of the hypertensive obese population of 221 individuals in Spain, using classical stepwise logistic regression and two Machine Learning methods, notably the Least Absolute Shrinkage and Selection Operator (LASSO) and the Elastic Net. The two Machine Learning approaches outperformed Logistic Regression in terms of AUC with 0.76 for Elastic Net and 0.74 for LASSO, against 0.64 scored by Logistic Regression. Even in terms of misclassification rate, Elastic Net turned out to be the best model, with 18% of misclassified data, against the 22% of LASSO and 25% of Logistic Regression, highlighting the importance of using proper Machine Learning models to improve the prediction of vitamin D regression in such cohorts [242]. Those studies, and many more, have proven the importance of using AI-based approaches in clinical and research questions around vitamin D levels, with a proper scientific justification for their usage in the predictive perspective around their application in supporting the clinicians and the caregivers picking up the best strategies for vitamin D supplementation, not just at the cohort level, but even at a personal level of the single individual or patient.

### 5.2. Toward Enhancing Acceptability: Sensory Analysis and “Functional Foods”

Scientific research, particularly that which focuses on neuroscience, has fully entered the consumer domain through the applications of neuromarketing and similar concepts. This approach is also currently popular in the field of healthcare and well-being, with the majority of companies investing a lot in the identification and implementation of convenient packaging to make their products more appealing from a (multi-)sensory and emotional perspective for the end-users, both at a citizen or patient level. In fact, the success of a given product also largely relies on its packaging and branding, and this also happens strongly with edible compounds, as demonstrated in [243]. When dealing with foods, the main sensory cues to be delivered to the consumer are represented by olfactory and gustatory features, along with a proper visual presentation, significantly affecting the pleasantness of a product and eventually determining its success or failure [244]. To this end, a mixed approach, merging the potentialities and advantages of instrumental, non-destructive methods [245] with sensory analysis, is key to building up novel edible compounds, which are, at the same time, valuable from a nutritional point of view and appealing in terms of packaging and presentation fashion for the consumer [14,246]. Also, sensory analysis should take advantage of traditional and innovative approaches, with the application of technology to detect implicit variables tied up with the emotional status of panelists able to complete the explicit methods traditional sensory analysis relies on [247,248].

**Figure 4:**
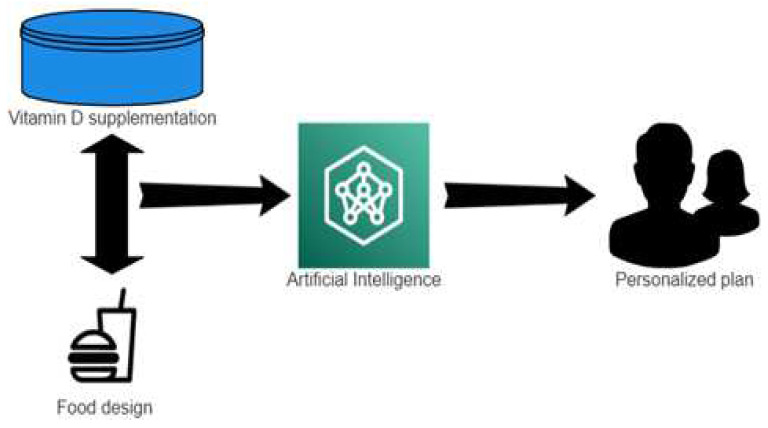
Simplified pipeline involving Artificial Intelligence and novel sensory analysis to draw up a personalized vitamin D supplementation plan.

Comprehensively, this approach should be carefully considered when designing and implementing new foods and, eventually, supplementation vectors to make vitamin D assumption viable and appealing from a sensory point of view, thus maximizing the treatment compliance even in subjects with peculiar sensory preferences or eventual disturbances possibly affecting sensory processing of external stimuli (Figure 4).

## 6. Conclusions

The tight correlation between AITD and CeD has been largely supported by common pathogenetic processes involved in these two conditions, including dysbiosis of the intestinal microbiota and impairment of the immune system, with vitamin D potentially exhibiting a crucial role in this complex interplay. Evidence from the literature suggests the association between vitamin D deficiency or insufficiency with an increased rate of HT and GD, while, for CeD, the results are somewhat controversial, with some research reporting that celiac patients do not significantly differ in vitamin D status compared to healthy controls. Importantly, most observational studies so far performed have a cross-sectional or case–control design, which does not allow definitive conclusions to be drawn about the causal relationships between vitamin D levels and the onset of AITD or CeD. Although current recommendations on vitamin D supplementation are based on skeletal health and do not address specific needs for the proper functioning of the immune system, findings from the reviewed studies indicate that an adequate intake of vitamin D might be beneficial in preventing or treating immune abnormalities such as HT, while for GD, results are more conflicting, and treatment with vitamin D does not significantly prevent GD relapse. As regards CeD, the actual role of vitamin D supplementation remains to be fully clarified, as well as the possible influence of GFD treatment on vitamin D levels. Prospective multicenter studies conducted in low-income countries that evaluate a greater number of variables (BMI, UV light exposure, dietary habits), as well as randomized controlled trials with a longer follow-up period, more accurate control of supplementation compliance, and possibly a larger number of subjects involved, are therefore warranted to establish the causal relationship between vitamin D status, AITD, and CeD. This would enable the identification of potential factors influencing 25(OH)D3 concentration and provide more reliable insights into the effectiveness of vitamin D supplementation as a therapeutic choice. Furthermore, establishing universally recognized normal vitamin D values would allow us to overcome certain limitations between studies and would indicate clear recommendations for monitoring vitamin D levels in patients with AITD or CeD, also from the perspective of primary prevention. Alternatively, personalizing vitamin D supplementation (even by functional foods, appealing from a sensory perspective) through AI would represent a powerful option for maximizing the outcome, reducing the risks associated with hypovitaminosis or, on the other side, intoxication, possibly linked to wrong vitamin D supplementation plans. Ultimately, a thorough understanding of the prevalence and risk factors of nutritional deficiencies, especially those related to vitamin D levels, during follow-up while on a GFD should represent an essential part of CeD management to prevent clinical consequences of nutritional imbalances.

## Figures and Tables

**Table 1:** Summary table reporting the guidelines on serum 25(OH)D3 levels and adequate intakes of vitamin D in the population.

Organization	Guidelines on Serum 25(OH)D3 Levels	Intake Recommendations
Endocrine Society	Deficiency: ≤20 ng/mL (50 nmol/L) Insufficiency: 21–29 ng/mL (52.5–72.5 nmol/L) Sufficiency: ≥30 ng/mL (72.5 nmol/L)	400 IU/day (10 µg): up to 12 months 600 IU/day (15 µg): ≥12 months-70 years old 1200–1800 IU/day (30–45 µg): adults with BMI > 30
European Food Safety Authority	Sufficiency: ≥20 ng/mL (50 nmol/L)	400 IU/day (10 µg): up to 12 months 600 IU/day (15 µg): ≥12 months-70 years old 800 IU/day (20 µg): ≥70 years old
Institute of Medicine	Sufficiency: ≥20 ng/mL (50 nmol/L)	400 IU/day (10 µg): up to 12 months 600 IU/day (15 µg): ≥12 months

Abbreviations: 25(OH)D3: 25-hydroxyvitamin D; BMI: body mass index.

**Table 2:** Clues and pitfalls in the association between levels of vitamin D and risk of autoimmune thyroid diseases.

Clues	Reference	Pitfalls	Reference
Significantly lower serum levels of 25(OH)D3 in patients with AITD as a whole, HT, and GD than in healthy controls.	[29,173,174,175,176]	No significant difference in 25(OH)D3 levels between patients with HT or GD and healthy controls.	[175,195]
Polymorphisms of *VDR* gene significantly associated with AITD or GD risk	[177,178,179]	High heterogeneity between studies due to variability of vitamin D assays, different cut-off values for vitamin D deficiency, and seasonal variations.	[29,173,174,175]
Vitamin D supplementation was able to significantly decrease Th17/Th1 ratio in patients with HT compared to the placebo group.	[185]	Only a few prospective studies and randomized controlled trials were performed.	[179,186,192,193,196]
Negative correlation between 25(OH)D3 levels/vitamin D supplementation and TPOAb levels in HT.	[187,188,192,193]	Lack of studies in some world areas and on participants with an African ethnicity.	[174]
Significant association of vitamin D supplementation and decreased levels of TGAb in HT.	[192]	Individual information on sun exposure, skin phototype, and dietary habits was missing in some studies.	[174,175]
Serum 25(OH)D3 concentration negatively correlated with serum IL-21 levels in both HT and GD patients.	[188]	Inconsistent results between the association of specific *VDR* gene polymorphisms and risk of AITD.	[177,178,179]
Significant lower levels of 25(OH)D3 in TSHRAb-positive GD patients compared to healthy controls or TSHRAb-negative patients.	[196]	No significant differences in vitamin D levels between different VDR genotypes.	[179]
Serum 25(OH)D3 levels at the time of ATD discontinuation associated with a higher incidence of GD recurrence.	[197]	Conflicting results on the association between vitamin D status and TPOAb levels in HT.	[187,188,189,190,191,192,193]
Weak negative correlation between 25(OH)D3 levels and TBII in GD patients with vitamin D deficiency	[186]	Positive correlation between vitamin D and IL-17, TNF-α, and IL-5 in HT patients.	[195]
Serum 25(OH)D3 levels were significantly lower in GD patients without remission compared to those with active disease.	[198]	No significant association between 25(OH)D3 levels and fT3, fT4, TSH, TPOAb, TSHRAb, or TGAb in GD patients.	[179,196]
		No significant association between 25(OH)D3 levels and Graves’ ophthalmopathy at diagnosis or with recurrence of GD after discontinuation of ATD.	[179]
		Serum 25(OH)D3 levels at the time of ATD discontinuation were not associated with TSHRAb or TBII in GD patients.	[197]
		Vitamin D supplementation was not significantly associated with a decrease in GD recurrence in patients with vitamin D deficiency.	[186]

Abbreviations: 25(OH)D3: 25-hydroxyvitamin D; AITD: autoimmune thyroid diseases; ATD: anti-thyroid drugs; BMI: body mass index; fT3: free triiodothyronine; fT4: free thyroxine; GD: Graves’ disease; HT: Hashimoto’s thyroiditis; IL: interleukin; TBII: TSH-binding inhibitory immunoglobulins; TGAb: anti-thyroglobulin antibodies; Th: T helper cell; TNF-α: tumor necrosis factor alpha; TPO: anti-thyroperoxidase antibodies; TSH: thyroid-stimulating hormone; TSHRAb: anti-thyroid-stimulating hormone antibodies; VDR: vitamin D receptor.

**Table 3:** Clues and pitfalls in the association between levels of vitamin D and risk of celiac disease.

Clues	Reference	Pitfalls	Reference
Presence of a north–south gradient—different degrees of exposure to sunlight associated with the onset of CeD.	[208,211]	Limited number of studies and with a cross-sectional design.	[213]
Gastrointestinal infections and rotavirus as risk factors of CeD in children potentially associated with vitamin D deficiency.	[215,216]	Lack of prospective studies and randomized controlled trials.	[214]
Decreased serum level of 25(OH)D3 more prevalent in CeD patients.	[213,214]	High heterogeneity between studies.	[214]
Higher tTG IgA in subjects with vitamin D deficiency compared to those with higher vitamin D levels.	[218]	No significant differences in 25(OH)D3 concentration in maternal blood from mid-pregnancy, postpartum, and cord plasma between children developing CeD and controls.	[217]
Significant increase in 25(OH)D3 levels following a GFD treatment, regardless of the vitamin D levels at the onset of disease.	[214,223]	Vitamin D deficiency was also detected in subjects on a long-term GFD.	[225,226]
Lower serum 25(OH)D3 in untreated CeD subjects compared to subjects on a GFD.	[234]	No association between 25(OH)D3 or 1,25(OH)_2_D3 deficiency and low bone mineral density.	[234]
		Elevated plasma levels of 1,25(OH)_2_D3 in untreated celiac patients.	[227,228]
		No normal values of vitamin D were reached in patients with vitamin D deficiency and treated with GFD.	[223]
		Vitamin D supplementation was not associated with a decreased risk of CeD or associated with an increased risk of CeD.	[235,236]

Abbreviations: 1,25(OH)_2_D3: 1,α,25-dihydroxyvitamin D3; 25(OH)D3: 25-hydroxyvitamin D3; CeD: celiac disease; GFD: gluten-free diet; Ig: immunoglobulin.

## Abbreviations

1,25(OH)_2_D3	1,α,25-dihydroxyvitamin D3
25(OH)D3	25-hydroxyvitamin D3
7-DHC	7-dehydrocholesterol
AhR	Aryl hydrocarbon
AI	Artificial Intelligence
AITD	Autoimmune thyroid diseases
AMP	Antimicrobial peptide
ANN	Artificial Neural Networks
APC	Antigen-presenting cell
BMI	Body mass index
CeD	Celiac disease
CTLA4	Cytotoxic T-lymphocyte-associated antigen 4
DC	Dendritic cell
fT3	Free triiodothyronine
fT4	Free thyroxine
GALT	Dut-associated lymphoid tissue
GD	Graves’ disease
GFD	Gluten-free diet
HLA	Human leukocyte antigen
HT	Hashimoto’s disease
IFN-γ	Interferon gamma
Ig	Immunoglobulin
IL	Interleukin
LXR	Liver X receptor
LPS	Lipopolysaccharides
MAPK	Mitogen-activated protein kinase
NF-κB	Nuclear factor kappa-light-chain-enhancer of activated B cells
NIS	Sodium/iodine symporter
NOD	Nucleotide-binding oligomerization domain leucine-rich repeats-containing receptors
PAMP	Pathogen-associated molecular patterns
PPARγ	Proliferator-activated receptor gamma
PRR	Pattern recognition receptor
PTH	Parathyroid hormone
ROS	Reactive oxygen species
SCFA	Short-chain fatty acid
TBII	Thyroid-stimulating hormone-binding inhibitory immunoglobulins
TGab	Thyroglobulin antibodies
TGF-β1	Transforming growth factor beta 1
Th cell	T helper cell
TLR	Toll-like receptor
TNF-α	Tumor necrosis factor alpha
TPOAb	Thyroid peroxidase antibodies
Treg	Regulatory T cells
TSHRAb	Thyroid-stimulating hormone receptor antibodies
tTG	Tissue transglutaminase
VDR	Vitamin D receptor
